# ‘It’s less traumatic because you’re in your own home’: exploring trauma-informed care for digital sexual health services – a secondary qualitative data analysis

**DOI:** 10.1136/sextrans-2024-056442

**Published:** 2025-06-24

**Authors:** Analisa Conway, Jo Gibbs, Tommer Spence, Alison Howarth, David Reid, Claudia S Estcourt, Fiona Burns, Karen C Lloyd

**Affiliations:** 1 Institute for Global Health, University College London, London, UK; 2 Department of Applied Health Research, UCL, London, UK; 3 Glasgow Caledonian University School of Health and Life Sciences, Glasgow, UK

**Keywords:** SEXUAL HEALTH, Gynecology, Diagnostic Screening Programs, PUBLIC HEALTH, QUALITATIVE RESEARCH

## Abstract

**Objectives:**

Trauma—an event or circumstance causing an individual physical and/or emotional harm—is associated with adverse sexual and reproductive health outcomes, including a higher prevalence of sexually transmitted infections. Trauma-informed care (TIC) is a systematic framework that recognises and addresses the impact of trauma through an organisation’s policies, practices and environment. Online delivery of sexual health services has rapidly become a standard of care in England; therefore, our research aims to provide valuable insights for implementing TIC in digital platforms.

**Methods:**

We performed a secondary analysis of qualitative data from two mixed methods studies that conducted semi-structured interviews with n=100 and n=25 sexual health service users following purposive sampling. A sample of 11 transcripts was included, and an inductive–deductive approach was used to analyse the data.

**Results:**

Our findings highlight six key themes of TIC: (1) Safety, (2) Trust and Transparency, (3) Peer Support and Self-Help, (4) Collaboration and Choice and (5) Cultural, Historical and Gender Issues. Participants reported that online postal self-sampling offered more privacy, comfort and control than in-person testing. They appreciated the use of gender-inclusive language and identified online postal self-sampling as a ‘safer option’ for individuals who fear being misgendered in clinical settings. However, some were concerned about providing sensitive information online, such as information about sexual partners or gender identity. There was limited evidence of peer support, and participants recommended improved signposting to sexual assault reporting and other trauma-related resources.

**Conclusions:**

This is the first known qualitative study exploring the intersection between TIC and digital sexual health interventions. Our study provides insight into how current online postal self-sampling practices facilitate the principles of TIC and which gaps remain. Future research should explore how these principles can be adapted to make digital sexual health services more trauma-informed.

**WHAT IS ALREADY KNOWN ON THIS TOPIC**

- Trauma-informed care (TIC) frameworks have not been explicitly applied to the development of digital sexual health services, despite the rising use of online postal self-sampling for sexually transmitted infection testing.

**WHAT THIS STUDY ADDS**

- Service users described how online postal self-sampling facilitated feelings of safety, control, and inclusion, which was particularly valued by those who had past negative experiences in clinic and among transgender and non-binary individuals. Gaps remain in implementing peer support and improving referral systems in online sexual health services.

**HOW THIS STUDY MIGHT AFFECT RESEARCH, PRACTICE OR POLICY**

- Further research should determine how the principles of TIC can be better adapted for digital health interventions.

## Introduction

The US Substance Abuse and Mental Health Services Administration (SAMHSA) defines trauma as ‘an event, series of events, or set of circumstances that is experienced by an individual as physically or emotionally harmful or life-threatening and that has lasting adverse effects on the individual’s functioning and mental, physical, social, or spiritual well-being’.^1^ A nationally representative survey of English residents found that nearly half of the population is exposed to at least one adverse or traumatic experience in childhood.^2^ Exposure to trauma is associated with poor sexual and reproductive health outcomes, including greater rates of sexually transmitted infections (STIs), unintended pregnancy and chronic pelvic pain.^3 4^ Sexual healthcare can be physically and situationally triggering for individuals with histories of trauma, thereby negatively impacting health-seeking behaviours, such as leading to avoidance of cervical screenings.^5^

The high prevalence of trauma and potential for retraumatisation underscores the need for sexual health services to operate with an understanding of and responsiveness to trauma. SAMHSA defines trauma-informed care (TIC) as a framework that ‘…realizes the widespread impact of trauma…recognizes the signs and symptoms of trauma in clients, families, [and] staff…and responds by fully integrating knowledge about trauma into policies, procedures, and practices, and seeks to actively resist re-traumatization’.^1^ TIC is a systemic approach informing organisational culture and practice through a ‘universal precaution lens’, meaning the assumption that every person could have a lifetime experience of trauma, regardless of disclosure.^1^ Following SAMHSA’s framework, there are six key principles of TIC described in table 1.

**Table 1:** Trauma-informed care principles^1^

Principle	Description	Example
Safety	The organisation provides an environment that ensures physical safety and promotes emotional safety through interpersonal interactions.^1^ This is reflected in safeguarding procedures and practices that aim to avoid retraumatisation.^26^	Healthcare providers may practise calming techniques, such as deep breathing, or play relaxing music, when patients appear or signal that they are distressed.
Trust and Transparency	The organisation is transparent about operations and decisions, and providers seek to build trusting relationships with service users.^1^ Trust and transparency can be accomplished by maintaining appropriate boundaries and providing clear information on service provision.^27^	If a patient expresses a preference for narration, the provider can describe what they are doing and why as they perform the examination.
Peer Support and Self-Help	The organisation offers or facilitates access to formal and/or informal networks of peers who share similar experiences.^1^	Providers should provide information on and referral to formal peer support group networks, such as organisations that support individuals who have experienced domestic abuse.
Collaboration and Mutuality	The organisation seeks to level power differences between providers and service users through building relationships and engaging service users in service delivery.^1 26^	Offer mechanisms for feedback (such as a survey) so that service users can voice their opinions and preferences regarding service delivery.
Empowerment, Voice and Choice	The organisation emphasises shared decision-making and provides service users with choices throughout service delivery. A strength-based approach is adopted to give service users a sense of control and empowerment.^1^	Provide patients with choices throughout their care; for example, giving patients the choice to shift clothing out of the way, rather than changing into a gown.
Cultural, Historical and Gender Issues	The organisation confronts stereotypes and biases related to race, ethnicity, sexuality, age, gender identity, religion and other identities. It also considers cultural context, offers gender-responsive services and is responsive to individuals’ needs.^1^	Ensure that a translator is available for patients that need interpretation services to understand the informed-consent process and to ask questions.

England has experienced a rapid shift to remote consultations and self-sampling for STIs due to pressure on services stimulated by restricted funding, increased demand and the COVID-19 pandemic.^6 7^ Online consultations accounted for a greater proportion (48%) of STI testing among London residents than face-to-face consultations (45%) in 2021.^8^ This is likely due to the scaling up of online postal self-sampling through the London Sexual Health Programme, a public health collaboration between the London boroughs. While digital health interventions can help remove barriers to care,^9^ there could be unanticipated consequences such as disrupting the patient–provider relationship or triggering trauma in service users.^10^

Although TIC guidelines have shaped the development and implementation of health service delivery since the early 2000s,^11^ they have not, to our knowledge, been explicitly applied to the development of digital health services. Therefore, the unique considerations for implementing TIC in a digital context are poorly understood. By drawing on qualitative data from two studies, the aim of this paper is to explore how service users’ beliefs about and experiences with online postal self-sampling can inform the implementation of TIC in online sexual health platforms. Findings from this research will identify gaps and highlight recommendations for making digital sexual health services more trauma-informed.

## Methods

We conducted a secondary analysis of qualitative data from two National Institute for Health and Care Research (NIHR)-funded studies, (1) ASSIST (NIHR129157)^12^ and (2) SEQUENCE Digital (NIHR200856). ASSIST (295506) and SEQUENCE Digital (299331) were both approved by National Health Service ethics. Table 2 provides an overview of the data used for the current study, which only pertains to the service user qualitative interview components of both studies.

**Table 2:** Study characteristics

	ASSIST	SEQUENCE Digital
Objective	Mixed methods study evaluating the implementation of online postal self-sampling and its impact on access to sexual and reproductive health services, health inequities and clinical and economic outcomes.	Mixed methods research programme to design, develop and evaluate an eSexual Health Clinic. A pretrial qualitative study was conducted to explore the design of online partner notification for STIs.
Setting	Three case study areas: Birmingham, London and Sheffield.	Four NHS trusts located in London, Sheffield, North Devon, South Devon and one health board in Glasgow, Scotland. Plus, targeted online community recruitment of sexual health services within the UK.
Population	Sexual health service users of clinic–based services and/or online postal self-sampling.	Sexual health service users or both clinic-based services or online postal self-sampling.
Recruitment and sampling	Service user participants were recruited by healthcare professionals on the day of their consultation or by following a link on the clinic or online postal self-sampling website. Purposive sampling was used to over-represent gender minorities, men who have sex with men, people of colour and young people.	Purposive sampling of NHS service users (1) recruited by healthcare professionals on the day of their clinic consultation or during follow-up contact, and (2) targeted community recruitment was used to identify gender diverse participants through online methods and self-referral through an existing research network.
Inclusion criteria and participants	People aged ≥16 years old who have used online and/or face-to-face services in any of the case study areas within the past 12 months. n=100 participants.	People aged ≥16 years old (or 18+ in Scotland), living in the UK, having access to a phone or the internet, being sexually active and either being diagnosed with a bacterial STI or in contact with a sexual partner who has been diagnosed with a bacterial STI in the past 6 months. n=25 participants.
Data collection and storage	Remote (phone or video conference) or face-to-face semistructured interviews. Interviews were audio recorded, pseudonymised by assigning a study ID and identifiers removed, and transcribed verbatim. Audio recordings were deleted after transcripts were checked for accuracy.	Remote semistructured interviews (phone or video conference). Interviews were audio recorded, pseudonymised by assigning a study ID and identifiers removed and transcribed verbatim. Audio recordings were deleted after transcripts were checked for accuracy.

NHS, National Health Service; STIs, sexually transmitted infections.

### Analysis

Researchers from ASSIST (TS, DR) and SEQUENCE Digital (KCL) identified a subsample of interviews (n=28) out of the n=125 from the two studies, including those with topics related to TIC and trauma, though trauma was not explicitly mentioned in topic guides. Transcripts were read by AC and included in analysis if they had themes related to TIC and/or discussion of how previous trauma or stigma has impacted sexual healthcare or preferences. This narrowed the sample to 11 transcripts.

An inductive–deductive approach to Braun and Clarke’s reflexive thematic analysis^13^ was used to analyse the data. AC and KCL read transcripts to become familiar with the data, taking note of initial trends. A preliminary round of inductive coding was conducted using the Microsoft Word ‘comments’ function. Following this initial round, AC performed several iterations of coding using Nvivo12, organising final codes into SAMHSA’s TIC framework.^1^ We combined the principles ‘collaboration and mutuality’ and ‘empowerment, voice, and choice’ into one category, termed ‘collaboration and choice’, due to overlapping definitions and examples in the data. AC, JG, TS, AH, DR and KCL met several times to review the coding process and discuss findings.

### Reflexivity

This research was conducted by an interdisciplinary team including a global health master’s student, clinicians and researchers with backgrounds in medical sociology, applied health sciences, epidemiology and sexual health. Reflexive practices included writing memos or notes of our thoughts while coding the data and discussing our own unique interpretations with the team, allowing us to acknowledge subjectivity in the research while staying true to the participant’s voices.

## Results

### Participant characteristics

Table 3 summarises the demographic data for the ASSIST and SEQUENCE Digital participants included in the analysis. Most participants were in the ≤24 and 25–34 age categories, while only two participants were 35 or older. Five participants identified as female, four as non-binary and two as male. Additionally, four participants identified as transgender or as not having the same gender as the sex they were assigned at birth. All participants reported their ethnicity as white British/white other. More than half of the sample described themselves as bisexual, gay or lesbian.

**Table 3:** Participant demographics

	ASSIST (n=7)	SEQUENCE Digital (n=4)	Total (n=11)
Age			
≤24	4	0	**4**
25–34	2	3	**5**
35–44	1	0	**1**
45–54	0	1	**1**
55–64	0	0	**0**
65+	0	0	**0**
Gender			
Female	3	2	**5**
Male	2	0	**2**
Non-binary	2	2	**4**
Other	0	0	**0**
Identifies as trans			
Yes	2	2	**4**
No	4	2	**6**
Prefer not to say	1	0	**1**
Ethnic group			
Asian/Asian British	0	0	**0**
Black/African/Caribbean/black British	0	0	**0**
Mixed/multiple ethnic groups	0	0	**0**
White British/white other	7	4	**11**
Other	0	0	**0**
Prefer not to say	0	0	**0**
Sexual orientation			
Bisexual	2	1	**3**
Gay or lesbian	3	1	**4**
Heterosexual or straight	1	2	**3**
Other	1	0	**1**
Prefer not to say	0	0	**0**

### TIC principles in online postal self-sampling delivery

Participants described their beliefs about and experiences of using online postal self-sampling, revealing how digital services could facilitate TIC, which gaps remain, and recommendations for improvement. SAMHSA’s TIC framework has been adapted to organise findings into the following principles: (1) safety, (2) trust and transparency, (3) peer support and self-help, (4) collaboration and choice and (5) cultural, historical and gender issues.

#### Safety

Responses focused mostly on how at-home testing limited participants’ distress and increased overall comfort. One participant shared:

…it’s less traumatic because you’re in your own home…you could play music, you could be maybe a bit more relaxed… (non-binary person, age 20–24)

Several participants described online postal self-sampling as more private and ‘anonymous’ than face-to-face testing. This was facilitated by the discreet delivery of the sampling kits, for example:

…I was really happy with [the packaging] because obviously it’s not something you want your family members if you live with them seeing, or your friends…it might not be something you want to share with people. (cis woman, age <20)

This greater sense of privacy was especially significant for individuals who had previous negative experiences with face-to-face testing due to perceived stigma, invasive questions surrounding gender identity and feelings of stress or embarrassment. One participant shared that:

…walking into an actual sexual health clinic can be a bit stressful or just an uneasy process. And I think if someone knows that they can do it in the comfort of their own house, I think that also kind of takes that element of maybe just like kind of embarrassment away. (cis woman, age 24–34)

#### Trust and transparency

Some participants were concerned about providing identifying and potentially sensitive data online. One participant highlighted that online postal self-sampling platforms should include clear and accessible information regarding data usage, storage and privacy to build trust with service users. They described their hesitancy to share their gender identity online, saying:

…I’d be cautious about where—to what degree different places get access to information that I’m trans…Because like—the concentration camps in the United States during World War II were set up on the basis of what’s called census data. (non-binary person, age 24–34)

To increase transparency, participants suggested that online questionnaires should include information boxes explaining why certain demographic data is collected. For example:

[the questionnaire] asks for like gender identity and asks who you have sex with….if there is an information button where you can ask… why are you asking this question? And then it answers you there… (non-binary person, age 20–24)

#### Peer support and self-help

Participants did not discuss experiences accessing formal peer support through online postal self-sampling. However, one participant described how he developed a form of peer support by completing the self-sampling kit along with his partner and flatmates. He said,

…it’s been an ongoing joke that the entire student flat I lived in would all order test kits at the same time and would all do the finger pricks together. (cis man, age 20–24)

Participants recommended more signposting to trauma-related resources and guidance for self-referral. This included adding contact information for sexual assault reporting and counselling, intimate partner violence helplines and other trauma-related resources so people can access what they need. One participant suggested:

…[including] an area dedicated to signpost people who’ve had traumatic sexual experiences to the right help…you can say stuff like, ‘Sex is often fun and exciting…but sometimes it isn’t and - it’s a very disturbing experience and we’re here to help support you through all the different scenarios. (cis woman, age 45–54)

They also pointed out services that facilitate online partner notification should recognise that some users experience non-consensual sexual encounters, including a message such as:

If you had a non-consensual encounter, we do not expect you to follow [online partner notification] up. Please speak to this person confidentially about this…then they could get signposted off to someone they can speak to. (cis woman, age 45–54)

#### Collaboration and choice

Participants’ responses detailed how online postal self-sampling gave them more choice and control over the STI testing process. Participants appreciated that they were able to do testing on their ‘own terms’ by completing the kit at a time that was most convenient for their schedule:

…you can do [the kit] in your own free time, if you want to do it when you get home from work, you can, if you want to do it at 3 am in the morning because you feel like it… (cis woman, age <20)

Receiving results over text or email is becoming the norm for remote and in-person STI testing. One participant said this form of communication contributed to feelings of control because she could choose when and where to access her results:

…having a text message or an email notification to say your results are now available, you can log into this page, or you can access it through this app, or whatever…it’s putting you in control of it and then being able to open up a page in whatever environment you want… (cis woman, age 24–34)

However, one participant had a stressful experience receiving results at work, which demonstrates how it could be beneficial for service users to have more options over how and when they receive their results:

I got the ‘Your kit has been tested, please look into your account or contact us on this number’…what was very stressful was dealing with the kind of aftermath of receiving a positive result while at work… (cis man, age 20–24)

Another described her preference for self-sampling because of its flexibility, allowing her to match aspects of online postal self-sampling service delivery to her needs. She chose to self-sample at home due to a prior misdiagnosis and painful swabbing experience at a clinic, but she did not feel comfortable completing the blood test on her own. The participant was able to tailor the service to her needs, doing the self-swabbing at home and the blood test at a clinic:

…The receptionist reassured me and said, ‘No, send your swab off. They can still test that. And put whatever blood is in the vial, and it will just go as inconclusive. And then come in, get your bloods done…’ (cis woman, age 20–24)

#### Cultural, historical and gender issues

Participants highlighted the use of inclusive language and awareness of trans and non-binary service users’ needs in their experience with online postal self-sampling. One participant who identifies as non-binary felt that it was a ‘mini affirmation’ to see their identity represented in the online form. Another participant shared:

I suppose that being someone who would describe themselves as transgender, I’m used to… having to describe myself in ways I’m not comfortable with in terms of my sex and gender…I recall on the website, it was much more broad in terms of how you described your gender. (trans woman, age 25–34)

Several participants described negative experiences in face-to-face settings, such as misgendering by staff, being asked inappropriate questions related to gender identity or feeling subjected to heteronormative assumptions.

…they’d assumed that I was cis and just started asking me questions about anatomy that I don’t have. I think the exact question was, ‘Are you having any pain in your testicles?’ And I said, ‘Well I don’t have testicles,’ and she looked at me like I’d just said I was, I don’t even know. (trans man, age 24–34)

Finally, negative experiences at in-person clinics led one participant to identify online postal self-sampling as a ‘safer’ option for individuals who are fearful of being misgendered.

## Discussion

To our knowledge, this is the first qualitative study exploring an evidence base for implementing TIC in digital sexual health services. Our findings suggest that online health services can promote feelings of safety, control and inclusion, particularly for those with past negative experiences in clinical settings. Online services also present unique considerations for service users, such as concerns about sharing private information online and limited opportunities for peer support. Overall, our analysis provides insight into how online postal self-sampling services reflect the principles of TIC and the gaps that remain.

Remote testing contributed to participants’ comfort and feelings of safety since they could engage in care from the privacy of their home, thereby avoiding the stigma and embarrassment that several associated with attending in-person clinics. This supports evidence that digital health interventions may help in overcoming stigma as a barrier to STI testing.^14–16^ Online postal self-sampling also offered participants greater flexibility and opportunity for choice; for example, one participant who had previously experienced painful swabbing at a clinic in the past engaged with the service to follow a ‘hybrid care’ option, doing the swabs at home and attending a clinic for the blood test. This example of personalised care aligns with literature suggesting that digital health services can be tailored to meet an individual’s unique and specific needs,^17^ which is an important aspect of TIC.

Findings related to the TIC principle ‘cultural, historical, and gender issues’ centred exclusively on gender among our sample, with nearly half of participants self-identifying as transgender or non-binary. Most notably, one participant described online postal self-sampling as a ‘safer option’ for individuals who are concerned about being misgendered in clinical settings. This is consistent with another study reporting that transgender and non-binary individuals expressed a preference for virtual care when they had previous experiences of stigma or misgendering in healthcare settings.^18^ It is unclear whether the perception of online postal self-sampling as a safer option is influenced more by the avoidance of face-to-face interactions with providers, or whether participants viewed the service as more inclusive in design. There was no discussion of how services can be informed from a race, ethnicity or cultural identity perspective. To date, most research on TIC has focused primarily on white or European/North American populations,^19^ revealing a gap in exploring the experiences of ethnic and racial minorities, Indigenous populations and service users in more global settings.

Findings also revealed gaps for implementing TIC in digital sexual health services. Although one participant described actively shaping their experience with online postal self-sampling through a hybrid care model, specific examples of the principle ‘collaboration and mutuality’ were otherwise absent from the data. This may be due to the design of online postal self-sampling, which limits interactions between service users and providers, unless it is necessary for treatment or if a follow-up appointment is requested. While limited interpersonal interaction with providers may be perceived as a benefit for some service users (for privacy reasons described above), it may also prevent users from providing meaningful feedback and shaping service delivery.

Participants did not discuss beliefs about or experiences of peer support in the delivery of online postal self-sampling. This lack of evidence could be due to individuals in the sample not wishing to disclose trauma or help-seeking behaviours, especially since they were not directly asked about these experiences in interviews. However, several participants suggested better signposting for service users to report sexual assault or intimate partner violence and access peer or professional support. A study evaluating sexual assault reporting among users of online sexual health services found a low reporting rate, with more than 75% of people who reported sexual assault claiming they did so in error or declining to engage in discussion during a follow-up phone call.^20^ Therefore, user understanding of e-triage questions and the possibility that some individuals wish to access support resources without direct clinical contact need to be taken into consideration.

Finally, participants expressed hesitancy to share identifying data online, especially related to gender identity or sexual partners, which is consistent with other studies.^21 22^ Patient mistrust in data sharing and privacy concerns have been identified as factors reducing the effectiveness and acceptability of digital mental health interventions.^23^ Participants suggested that online postal self-sampling should include accessible information on data privacy and that rationales should be provided when asking users to share sensitive data.

### Clinical implications

Our study highlights how digital sexual health services may serve as a more accessible and comfortable alternative to face-to-face testing for individuals who have experienced trauma. A key finding is the need for online services to improve sexual assault and intimate partner violence reporting and referral to support, especially given that receiving a follow-up phone call may not be acceptable to all service users.^20^ Developing user-controlled reporting mechanisms that allow users to choose whether they want follow-up, what kind of resources (eg, peer, clinical, informational) and through which mode (eg, email, text, call) could serve as a possible solution. However, several challenges must be addressed—most notably, ongoing concerns around the security of sharing sensitive information online and potential partner monitoring, in the case of intimate partner violence, which may limit the safety of using online tools.

### Strengths and limitations

One limitation is the use of secondary data since ASSIST and SEQUENCE Digital were not originally designed with the aim of exploring TIC. It is possible the sample is biased, for example, people who have experienced trauma may have been less likely to participate in the study. While the TIC principles offered a useful framework for analysis, it may have limited the findings since the principles have areas of overlap and definitions can vary in the literature. A strength of this exploratory study was the use of qualitative methods, which allowed for a nuanced understanding of individuals’ experiences and beliefs about using online postal self-sampling.

### Suggestions for future research

There is very limited research to date on TIC approaches for designing and delivering digital health interventions.^24^ This study demonstrates the need to bridge the gap between digital health service delivery and trauma-informed frameworks of care. Our findings highlight how service users’ beliefs and experiences of online postal self-sampling tie into the principles of TIC, but future research is needed to explore the application of these principles in digital sexual and reproductive health services more explicitly. For instance, more research is needed to understand the complexities behind individuals’ preferences regarding trauma disclosure and support seeking through digital platforms versus face-to-face settings.^25^

## Conclusion

Our study aimed to investigate sexual and reproductive health service users’ beliefs about and experiences of online postal self-sampling to better understand TIC approaches in relation to online services. Our findings suggest the discreet, flexible and inclusive design of online postal self-sampling facilitated feelings of safety and control, with one participant identifying it as a ‘safer option’ for transgender and non-binary individuals. However, concerns about sharing identifying information online and the need for improved systems to connect service users with trauma-related reporting tools and support indicate that gaps remain in making sexual health services trauma-informed. Future research should investigate how to adapt the TIC principles for online modalities of care to inform future guidelines for practice, service design and delivery.

## Data Availability

No data are available. The data that support the findings of this study are not publicly available as, due to the sensitive nature of the questions asked in these studies, participants were not asked and did not consent to this. Questions about the data can be directed to JG, co-chief investigator for ASSIST and co-investigator for SEQUENCE digital.
